# Conductive heating mattress leads to ECG changes that mimic pacemaker spikes

**DOI:** 10.1007/s10877-020-00520-z

**Published:** 2020-07-15

**Authors:** A. Bräuer, R. Franke, A. von Hammerstein-Equord

**Affiliations:** 1 Department of Anesthesiology, University Medical Center, Georg-August University, Robert-Koch-Str. 40, 37075 Göttingen, Germany; 2 Department of Thoracic and Cardiovascular Surgery, University Medical Center, Georg-August University, Robert-Koch-Str. 40, 37075 Göttingen, Germany

**Keywords:** Hypothermia, Heating mattress, Artefact

## Abstract

Hypothermia is a common perioperative complication. To prevent perioperative hypothermia amongst other things electrical heating mattresses are used. We have made an observation with the use of an electrical heating mattress that may confuse users. In this case the ECG monitoring suddenly showed spikes that looked like spikes from an implanted pacemaker. When turning off the heating mattress the spikes disappeared and returned after turning on the heating mattress again.

Hypothermia is a common perioperative complication that increases morbidity of the patients [1]. Therefore, guidelines all over the world recommend active warming therapy. Forced-air warming is the most important method for perioperative warming, but in many guidelines conductive warming methods are recommended as another form of active warming therapy [2]. Although the efficacy of active warming with conductive warming methods is still debated [3–5] it is used quite often.

We have made an observation with the use of an electrical heating mattress that may confuse users. In this case a patient undergoing Video Assisted Thoracic Surgery (VATS) in the lateral decubitus position was actively warmed from underneath using a UniqueTemp° Jelly blanket (Geratherm Medical AG, Geschwenda, Germany) and both legs were warmed by two leg blankets (UniqueTemp° Geratherm Medical AG, Geschwenda, Germany) after positioning on the operating table.

During surgery the ECG monitoring suddenly showed spikes that looked like spikes from an implanted pacemaker.

These spikes had no correlation to the QRS complex and the pattern looked like an ECG of a patient with pacemaker dysfunction, although the patient did not have an implanted pacemaker. When turning off the heating mattress the spikes disappeared (Fig. 1) and returned after turning on the heating mattress again.

**Fig. 1:**
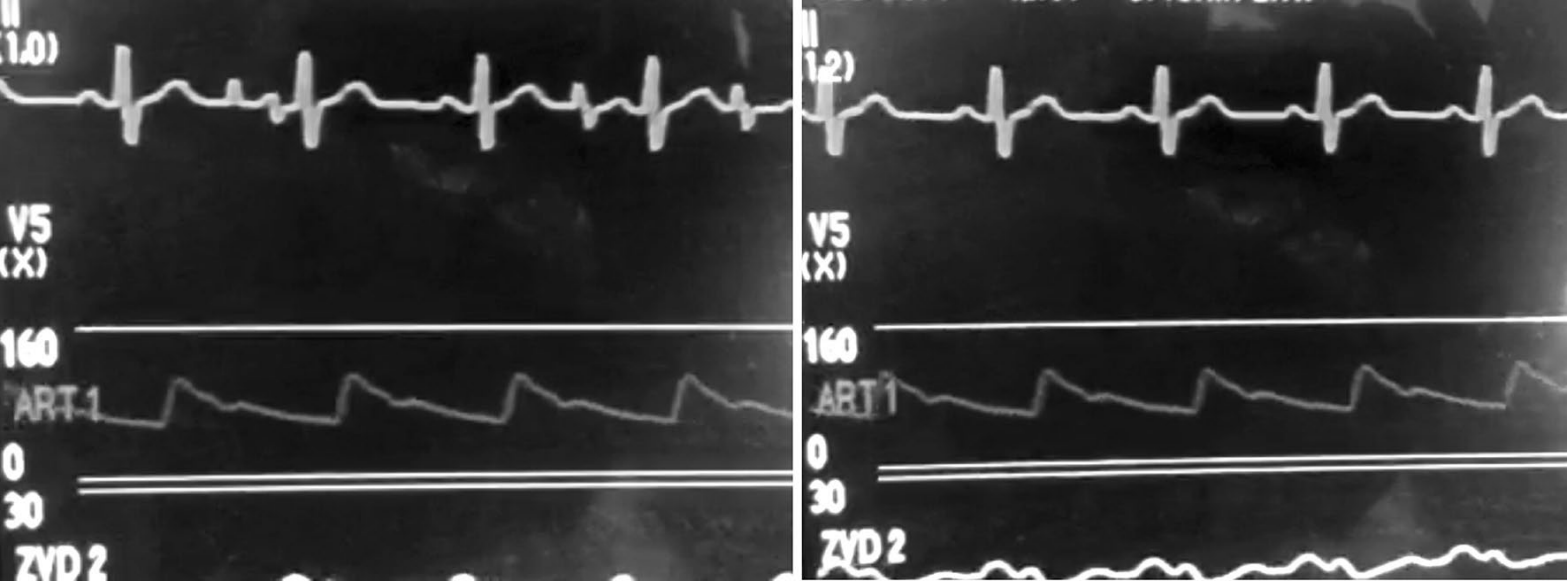
ECG and arterial blood pressure tracing of a screenshot from a smartphone video of the ECG with artefacts looking like pacemaker spikes an immediately after the heating mattresses were switched off

It seems that these spikes were caused by interference of the electrically driven conductive heating blankets with the ECG. The warming system uses carbon-fibers that are heated with a maximum of 35.5 V direct current by a control unit. These current-carrying carbon fibers or the control unit can generate an electromagnetic field that may have caused these artefacts. The artefacts may be falsely interpreted as pacemaker dysfunction in a patient that has a pacemaker implanted.

The device was controlled after use and no technical problems could be detected. The manufacturer claims that the device meets the requirements of Council Directive 93/42 EEC of 14.06.93 on Medical Devices and of the standard DIN EN 60601-1 (Medical electrical equipment, Part 1: General requirements for safety), DIN EN 60601-1-2 (Medical electrical equipment Part 1–2: General requirements for safety—Collateral standard: Electromagnetic compatibility—Requirements and Tests), DIN EN 12470-4 and DIN EN 60601-2-35 (Medical electrical equipment, Part 2: Particular requirements for the safety of blankets, pads and mattresses intended for the warming of patients in medical use) and that a DIN EN ISO 13485 certified quality management system ensures that these requirements are met and entitles the manufacturer to use the CE 0118 label.

In the instruction manual the manufacturer states “Do not place the control unit directly on top of ECG or EEG equipment. Otherwise, faults may arise, e.g. the devices may produce false readings.” However, we placed the control unit on an IV pole far away from the monitoring equipment. The monitoring system (Philips IntelliVue MX500, Philips Medizin Systeme Boeblingen GmbH, Boeblingen Germany) is mounted on the ceiling and had no direct or indirect contact to the control unit and or the heating mattress.

In our view users of the system should be aware of the possibility that an electrical heating mattresses can cause artifacts that look like pacemaker spikes even if all safety precautions given by the manufacturer are take into consideration.

## References

[1] Sessler DI (2016). Perioperative thermoregulation and heat balance. Lancet.

[2] NICE. Addendum to Clinical Guideline 65, Inadvertant Perioperative Hypothermia. 2016. https://www.nice.org.uk/guidance/cg65. Accessed 19 Mar 2020.

[3] Emmert A, Franke R, Brandes IF (2017). Comparison of conductive and convective warming in patients undergoing video-assisted thoracic surgery: a prospective randomized clinical trial. Thorac Cardiovasc Surg.

[4] Negishi C, Hasegawa K, Mukai S (2003). Resistive-heating and forced-air warming are comparably effective. Anesth Analg.

[5] Röder G, Sessler DI, Roth G (2011). Intra-operative rewarming with Hot Dog® resistive heating and forced-air heating: a trial of lower-body warming. Anaesthesia.

